# Treatment and Outcomes in Advanced Gastroesophageal Adenocarcinoma in the Pre-Immunotherapy Era Based on the Spanish AGAMENON-SEOM Registry

**DOI:** 10.3390/cancers17132164

**Published:** 2025-06-27

**Authors:** Paula Jimenez-Fonseca, Alberto Carmona-Bayonas, Jaime Álvarez-Cañada, Amy Storfer-Isser, Marta Martin-Richard, Tamara Sauri, Juana María Cano, Elia Martínez Moreno, Pablo Pérez-Wert, Javier López, Francisco Garcia Navalon, Lucía Gómez-González, Maribel Ruiz Martín, Ana Belén Rupérez Blanco, Flora López-López, Emilse Roncancio-Díaz, Belén Corbacho, Marta Mateo, Paloma Anguita-Alonso, Javier Gallego Plazas

**Affiliations:** 1 Medical Oncology Department, Hospital Universitario Central de Asturias, Instituto de Investigación Sanitaria del Principado de Asturias (ISPA), 33011 Oviedo, Spain; 2 Medical Oncology Department, Hospital Universitario Morales Meseguer, Instituto Murciano de Investigación Biosanitaria (IMIB), University of Murcia, 30008 Murcia, Spain; alberto.carmonabayonas@gmail.com (A.C.-B.); javiaguilas96@gmail.com (J.L.); 3 Astellas Pharma Spain, 28046 Madrid, Spain; jaime.alvarez@astellas.com (J.Á.-C.); belen.corbacho@astellas.com (B.C.); marta.mateo@astellas.com (M.M.); paloma.anguita@astellas.com (P.A.-A.); 4 Astellas Pharma, Inc., Northbrook, IL 60062, USA; amy.storfer-isser@astellas.com; 5 Medical Oncology Department, Catalan Institute of Oncology, 08908 L’Hospitalet, Spain; martamartin@iconcologia.net; 6 Medical Oncology Department, Hospital Clinic and Translational Genomics and Targeted Therapies in Solid Tumors IDIBAPS, 08036 Barcelona, Spain; sauri@clinic.cat; 7 Medical Oncology Department, Hospital General Universitario de Ciudad Real, 13005 Ciudad Real, Spain; juanamariacano@gmail.com; 8 Medical Oncology Department, Hospital Universitario de Fuenlabrada, 28942 Madrid, Spain; martinezmorenoelia@gmail.com; 9 Medical Oncology Department, Hospital Universitario La Paz, 28046 Madrid, Spain; pablopwert@gmail.com; 10 Medical Oncology Department, Hospital Universitario Son Llatzer, 07198 Mallorca, Spain; fcogarcianavalon@gmail.com; 11 Medical Oncology Department, Hospital General Universitario de Alicante, 03010 Alicante, Spain; luciagomezgonzalez1@gmail.com; 12 Medical Oncology Department, Hospital Universitario Rio Carrión, 34005 Palencia, Spain; mabelruizmar@yahoo.es; 13 Medical Oncology Department, Hospital Universitario de Toledo, 45007 Toledo, Spain; abelenruperezblanco@hotmail.com; 14 Medical Oncology Department, Hospital Universitario del Sureste, Arganda del Rey, 28500 Madrid, Spain; flora_989@hotmail.com; 15 Astellas Pharma Europe Ltd., Addlestone KT15 2NX, UK; emilse.roncancio-diaz@astellas.com; 16 Medical Oncology Department, Hospital General Universitario de Elche, 03203 Alicante, Spain; j.gallegoplazas@gmail.com

**Keywords:** adenocarcinoma, gastric neoplasms, gastroesophageal junction, HER2, neoplasms, survival

## Abstract

**Background/Objectives**: Human epidermal growth factor receptor 2–negative (HER2-negative), locally advanced or metastatic gastric/gastroesophageal junction adenocarcinoma (advanced G/GEJa) is associated with poor survival outcomes, and there is an unmet need for targeted therapy. This study, conducted in the pre-immunotherapy era, aimed to describe the characteristics and management, and compare the survival, of HER2-negative and HER2-positive patients initiating first-line (1L) treatment for advanced G/GEJa in Spain and estimate the number of HER2-negative patients eligible for 1L polychemotherapy. **Methods**: Patients from the AGAMENON-SEOM registry who initiated 1L polychemotherapy for advanced G/GEJa (2015–2019) in Spain were included. **Results**: In total, 1357 patients were included (951 [70.1%] HER2-negative; 315 [23.2%] HER2-positive; 91 [6.7%] unknown HER2 status). Most patients (56.3%) received one line of therapy; 27.6% received two lines; and 16.1% received three lines. Among HER2-positive patients, 92.7% received trastuzumab as part of 1L treatment. The use of FOLFOX and CAPOX increased over the study period (2015–2019). HER2-negative patients had significantly shorter progression-free survival (median, 5.92 months [95% CI, 5.59–6.38] vs. 7.37 months [95% CI, 6.55–8.29]; log-rank *p* < 0.0001) and overall survival (median, 10.49 months [95% CI, 9.74–11.05] vs. 13.82 months [95% CI, 12.30–14.74]; adjusted time ratio, 0.812 [95% CI, 0.722–0.913]; *p* = 0.0005) than HER2-positive patients. Per probabilistic sensitivity analyses, an estimated 2856 (95% CI, 1619–4134) Spanish patients with HER2-negative advanced G/GEJa were eligible for 1L polychemotherapy in 2024. **Conclusions**: The survival difference between HER2-positive and HER2-negative patients underscores the critical need for targeted therapies for HER2-negative patients in the 1L setting.

## Simple Summary

There is an unmet need for targeted therapy for human epidermal growth factor receptor 2–negative (HER2-negative), locally advanced or metastatic gastric/gastroesophageal junction adenocarcinoma (advanced G/GEJa), which is associated with poor survival outcomes. This study aimed to describe the characteristics, treatment, and survival of HER2-negative and HER2-positive patients with advanced G/GEJa in Spain. Among 1357 patients from the Spanish AGAMENON-SEOM registry who initiated first-line polychemotherapy for advanced G/GEJa between 2015 and 2019 (inclusive), 70.1% had HER2-negative disease. Overall, 56.3% of patients with advanced G/GEJa received only one line of therapy, and most (92.7%) HER2-positive patients received targeted therapy (trastuzumab) as part of first-line treatment. HER2-negative patients had significantly shorter progression-free survival (median of 5.92 months vs. 7.37 months) and overall survival (median of 10.49 months vs. 13.82 months) compared with HER2-positive patients. The survival difference underscores the critical need for targeted first-line therapies for HER2-negative patients.

## 1. Introduction

Gastric cancer is the fifth most common cancer worldwide and a leading cause of cancer-related death, with nearly a million new cases globally and more than 650,000 deaths in 2022 [1]. The worldwide age-standardized 5-year survival rate for stomach cancer was 20–40% in 2000–2014 [2], and patients with locally advanced or metastatic gastroesophageal junction (GEJ) adenocarcinoma have a median survival of 7–11 months [3]. The incidence rate of gastric adenocarcinoma is higher than that of GEJ adenocarcinoma; for example, in an analysis of real-world data from patients with advanced gastric, GEJ, or esophageal adenocarcinoma in the Spanish AGAMENON-SEOM registry, the primary tumor location was the stomach in the majority of patients (78%), compared with the GEJ (13%) or esophagus (9%) [4]. However, data demonstrate a decline in gastric adenocarcinoma and an increase in GEJ adenocarcinoma since the 1970s [1,5,6]. In Spain, gastric cancer was the ninth most common cancer in 2021 and the eighth most common cause of cancer-related death in 2020 [7]. Although mortality rates in Spain are lower than global rates, there are still over 5000 deaths in Spain every year due to gastric cancer [7], and the 5-year net survival rate between 2008 and 2013 was 27.4% among patients with gastric cancer [8]. The burden of gastric and gastroesophageal junction adenocarcinoma (G/GEJa) is high, even compared with other cancers, with increasingly severe symptoms, worsening health-related quality of life, and accelerating healthcare resource utilization and cost as the disease advances [9].

There have been therapeutic advances for patients with human epidermal growth factor receptor 2–overexpressing (HER2-positive) G/GEJa. Notably, trastuzumab is a monoclonal antibody that binds to the extracellular domain of the transmembrane receptor protein HER2, inhibiting proliferation of HER2-expressing tumors and activating antibody-dependent cellular cytotoxicity [10]. Trastuzumab, in combination with chemotherapy, significantly improved overall survival (OS) and progression-free survival (PFS) in patients with HER2-positive, locally advanced unresectable or metastatic G/GEJa (advanced G/GEJa) compared with chemotherapy alone in a clinical study [11] and subsequently became a standard treatment for this disease [12]. Prior to the advent of trastuzumab, patients with HER2-positive G/GEJa had outcomes similar to, or worse than, patients with HER2-negative disease [10,13,14]. However, they now have a survival advantage because of their eligibility for targeted therapy and trastuzumab deruxtecan as second-line (2L) therapy [13,15,16].

Subsequently, therapies targeting programmed death receptor 1 (PD-1), an immune checkpoint protein, became available. Pembrolizumab is a PD-1 inhibitor recently approved, in conjunction with trastuzumab and chemotherapy, for first-line (1L) treatment of patients with HER2-positive G/GEJa expressing programmed death-ligand 1 (PD-L1; combined positive score [CPS] ≥ 1) [17,18,19]. For patients with HER2-negative G/GEJa, pembrolizumab and nivolumab (another PD-1 inhibitor) are approved for 1L treatment of adults with HER2-negative advanced or metastatic G/GEJ or esophageal adenocarcinoma whose tumors express PD-L1 [17,18,20]. When used in combination with chemotherapy in patients with HER2-negative disease, nivolumab and pembrolizumab significantly improved PFS and OS compared with chemotherapy alone [21,22]. Because PD-L1 is not ubiquitously expressed in G/GEJ tumors (~50%) [23,24,25], there remains an unmet need for more effective, targeted treatment for patients with HER2-negative advanced G/GEJa who are not candidates for immunotherapy. Overall, approximately three-quarters of patients with advanced G/GEJa have HER2-negative disease [10,26].

This study was conducted using real-world data from the AGAMENON-SEOM registry to describe the clinical characteristics, treatment patterns, and survival outcomes (PFS and OS) in Spanish patients with advanced G/GEJa. Additionally, this study compared PFS and OS by HER2 status and estimated the number of patients in Spain with HER2-negative advanced G/GEJa who were eligible for 1L polychemotherapy.

## 2. Methods

### 2.1. AGAMENON-SEOM Registry

AGAMENON-SEOM is an observational, clinicopathological registry managed by the Spanish Society of Medical Oncology (SEOM). Its purpose is to describe the diagnosis and treatment approaches for G/GEJa in a clinical practice setting according to each participating center’s usual practice [27]. The registry contains data on over 4000 patients from 40 Spanish hospitals, including 2470 patients who received 1L polychemotherapy [4]. Sixteen of the 17 regions in Spain are represented, allowing geographical coverage across the country, with more participating centers located in regions with greater population density.

Eligibility criteria for patients included in this analysis of the AGAMENON-SEOM registry are summarized in Table 1. Briefly, eligible patients are adults (≥18 years) with histologically confirmed advanced gastric, GEJ, or distal esophageal adenocarcinoma who were treated with at least one cycle of 1L polychemotherapy and were followed for at least 3 months (except for patients who died sooner than 3 months after treatment initiation) [28,29,30]. Patients are excluded from the registry if they participated in a clinical trial without standard chemotherapy; received prior systemic therapy for advanced gastric, GEJ, or distal esophageal adenocarcinoma; completed prior neoadjuvant or adjuvant chemotherapy, radiotherapy, or chemoradiotherapy less than 6 months earlier; or were diagnosed with any cancer other than G/GEJa.

The data were gathered using a web-based data collection tool and managed through a website (http://www.agamenonstudy.com/) using filters and a system of queries to guarantee data reliability and control for inconsistent data [31].

### 2.2. Study Design

This longitudinal cohort study included patients from the AGAMENON-SEOM registry who initiated 1L polychemotherapy for advanced G/GEJa in Spain between 1 January 2015 and 31 December 2019 (Table 1). The study period was between 1 January 2015 and 31 December 2021, allowing at least 2 years of follow-up data for each patient. Patients were excluded from the study if they had distal esophageal cancer (primary site).

### 2.3. Objectives

Using this cohort of patients who received 1L polychemotherapy for advanced G/GEJa, the objectives of this study were (1) to describe the disease management for all patients and stratified by HER2 status; (2) to describe the sociodemographic and disease characteristics of all patients and stratified by HER2 status; (3) to compare PFS and OS by HER2 status for all 1L regimens, and OS for the subsets of patients who received 1L FOLFOX (folinic acid, fluorouracil, and oxaliplatin) or 1L CAPOX (capecitabine and oxaliplatin); and (4) to estimate the number of patients in Spain with HER2-negative advanced G/GEJa who were eligible for 1L polychemotherapy.

### 2.4. Statistical Analysis

Descriptive statistics were used to summarize disease management and patient sociodemographic and disease characteristics. PFS was defined as the time from the index date, defined as the initiation date of 1L polychemotherapy for advanced G/GEJa, until the date of progressive disease or date of death by any cause (whichever occurred first). OS was defined as the time from the index date to date of death by any cause. Patients were censored at the date of their last contact or the end of the study (whichever occurred first).

PFS and OS were summarized using Kaplan–Meier estimates. The log-rank test was used to compare PFS and OS for patients with HER2-positive disease and HER2-negative disease. *p*-values < 0.05 were considered statistically significant. Accelerated failure time (AFT) models with a generalized gamma distribution [32] were used to compare OS for patients with HER2-negative disease and HER2-positive disease, as the proportional hazards assumption was violated for HER2 status. AFT models were fitted without confounders (unadjusted) and with adjustment for confounders that were preselected by study investigators: Lauren histological subtype (intestinal; diffuse; mixed; not available/not classifiable), number of metastatic sites (0–3; ≥4), Eastern Cooperative Oncology Group performance status (ECOG PS: 0; 1; ≥2), presence of bone metastases (yes; no), presence of ascites (yes; no), and neutrophil-to-lymphocyte ratio (NLR) category (<4; 4 to <8; ≥8). The results from the AFT models included the coefficient, standard error, time ratio (TR), 95% confidence interval (CI), and *p*-value. A TR < 1 indicates that HER2-negative status was associated with shorter survival (accelerated failure) compared with HER2-positive status (the reference category).

Six parameters were required to estimate the number of patients in Spain with HER2-negative advanced G/GEJa who were eligible for 1L polychemotherapy. Since there is no single data source representative of the Spanish population that includes all six parameters, information from the literature and the results of this study were used. The values of each parameter and their data sources were as follows: the number of new cases of G/GEJ cancer in Spain in 2024 (6868 patients; parameter 1 [33]) with advanced disease (84%; parameter 2 [34]) that was adenocarcinoma (90%; parameter 3 [35]) who were eligible for polychemotherapy (78.7%; parameter 4 [30]), were tested for HER2 (93.3%; parameter 5 [results from this study]), and had HER2-negative disease (75.1%; parameter 6 [results from this study]). Probabilistic sensitivity analyses (PSAs) were conducted to account for the plausible variation across parameters 2–6. Each parameter was considered a random variable and was assumed to have a beta distribution. Parameters were simultaneously changed, with values drawn by random sampling from the beta distribution. The PSA was repeated 10,000 times to generate new parameter values, quantify the uncertainty of the parameters, and produce an average estimate and 95% CI.

## 3. Results

### 3.1. Disposition

Of 4479 patients with advanced G/GEJa assessed for participation in the AGAMENON-SEOM registry, 3315 patients fulfilled all eligibility criteria and 1164 patients were excluded for the following reasons: 1065 patients did not receive combination chemotherapy using at least two drugs, 50 patients were not followed for at least 3 months, and 49 patients had completed prior neoadjuvant or adjuvant chemotherapy, radiotherapy, or chemoradiotherapy less than 6 months earlier. Of the 3315 patients fulfilling all eligibility criteria for the registry, 1513 patients initiated 1L polychemotherapy for advanced G/GEJa between 1 January 2015 and 31 December 2019. After excluding 156 patients with distal esophageal adenocarcinoma, the final study population included 1357 patients.

### 3.2. Sociodemographic and Disease Characteristics

Of the 1357 patients with advanced G/GEJa who were included in this study, 951 (70.1%) patients had HER2-negative disease, 315 (23.2%) patients had HER2-positive disease, and 91 (6.7%) patients had unknown HER2 status (Table 2). The median age (range) of all patients at 1L treatment initiation was 64.7 years (19.9–88.5); most patients were male (67.3%), just under half were aged 65 years or older (48.9%), and just over half had a body mass index of 18.5 to <25 kg/m^2^ (53.9%). Most patients had an ECOG PS of 0–1 (85.5%), a primary tumor site of the stomach (71.6%), and de novo disease (84.5%). The proportions of patients with diffuse (38.5%) and intestinal (37.1%) histologies were similar. Just over half of patients had ≥2 metastatic sites (51.6%), and the most common sites were locoregional lymph nodes (63.3%), peritoneum (48.6%), and non-locoregional lymph nodes (44.2%). Metastatic ascites was reported in 23.6% of patients, and 11.6% of patients had bone metastases. The median (range) NLR was 3.4 (0.3–102.0). Most patients (60.4%) had a low NLR (<4); 27.3% had a moderate NLR (4 to <8); and 10.5% had a high NLR (≥8).

There were some notable differences between patients with HER2-negative disease and patients with HER2-positive disease in terms of sociodemographic and disease characteristics. Compared with patients with HER2-positive disease, patients with HER2-negative disease were slightly younger (median age, 63.8 vs. 65.8 years) and fewer were male (64.7% vs. 75.6%); additionally, their primary tumor site was more likely to be gastric (75.6% vs. 58.1%) and to have diffuse histology (45.4% vs. 18.4%).

### 3.3. First-Line Treatment

**Figure 1:**
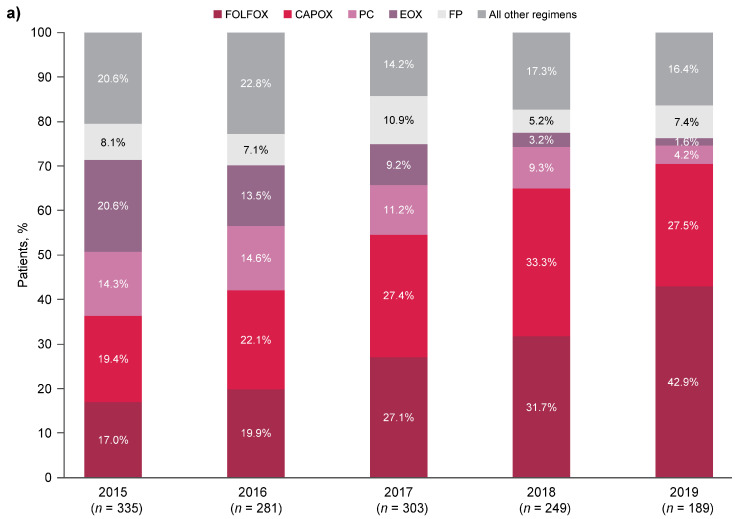

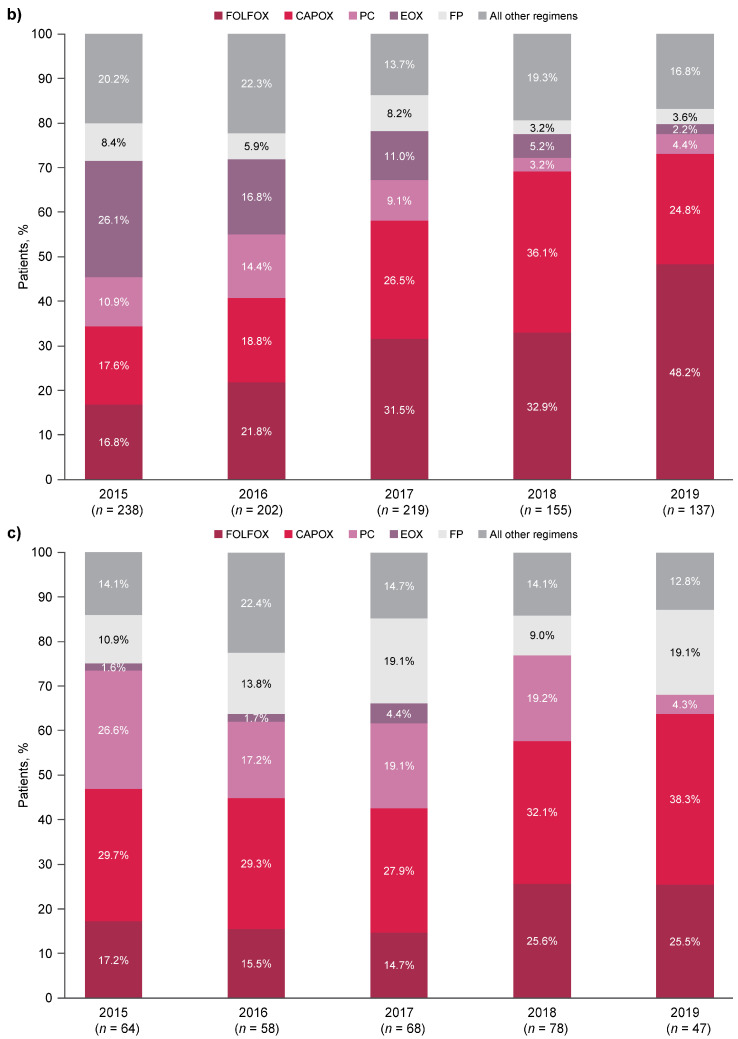
1L chemotherapy regimen by year of treatment initiation for (**a**) all patients, (**b**) patients with HER2-negative disease, and (**c**) patients with HER2-positive disease. 1L, first-line; CAPOX, capecitabine and oxaliplatin; EOX, epirubicin, oxaliplatin, and capecitabine; FOLFOX, folinic acid, fluorouracil, and oxaliplatin; FP, fluorouracil and cisplatin; HER2, human epidermal growth factor receptor 2; PC, cisplatin and capecitabine.

Most patients (56.3%) received only one line of therapy. Between 2015 and 2019, the median (interquartile range) duration of 1L treatment was 4.8 months (2.8–7.2), and patients received a median (interquartile range) of 6 (4–11) chemotherapy cycles during 1L treatment (Table 3). Of the 315 patients with HER2-positive disease, 292 (92.7%) received trastuzumab as part of their 1L treatment regimen. Use of FOLFOX and CAPOX increased during the 2015–2019 period, as these therapies became commonly used 1L regimens for advanced G/GEJa from 2017 onward (Figure 1a). In the overall population and HER2-negative subgroup, the most common 1L chemotherapy regimen in 2019 was FOLFOX (Figure 1a,b); CAPOX was the most common 1L chemotherapy regimen in 2019 for patients with HER2-positive disease (Figure 1c).

### 3.4. Second- and Third-Line Treatment

**Figure 2:**
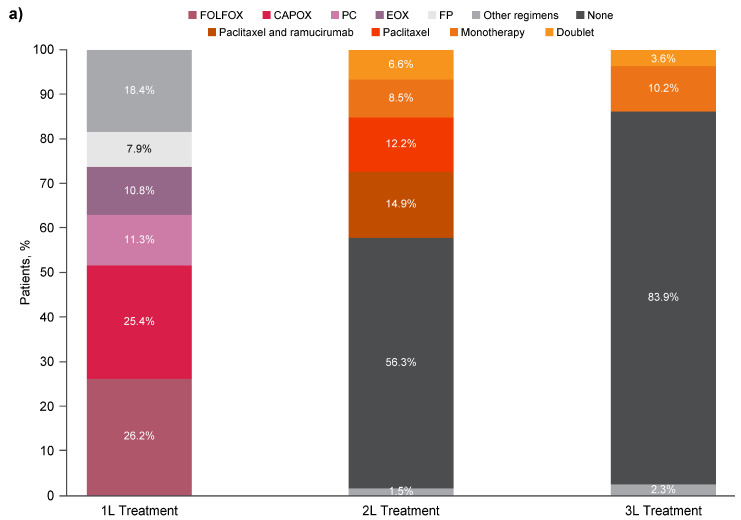

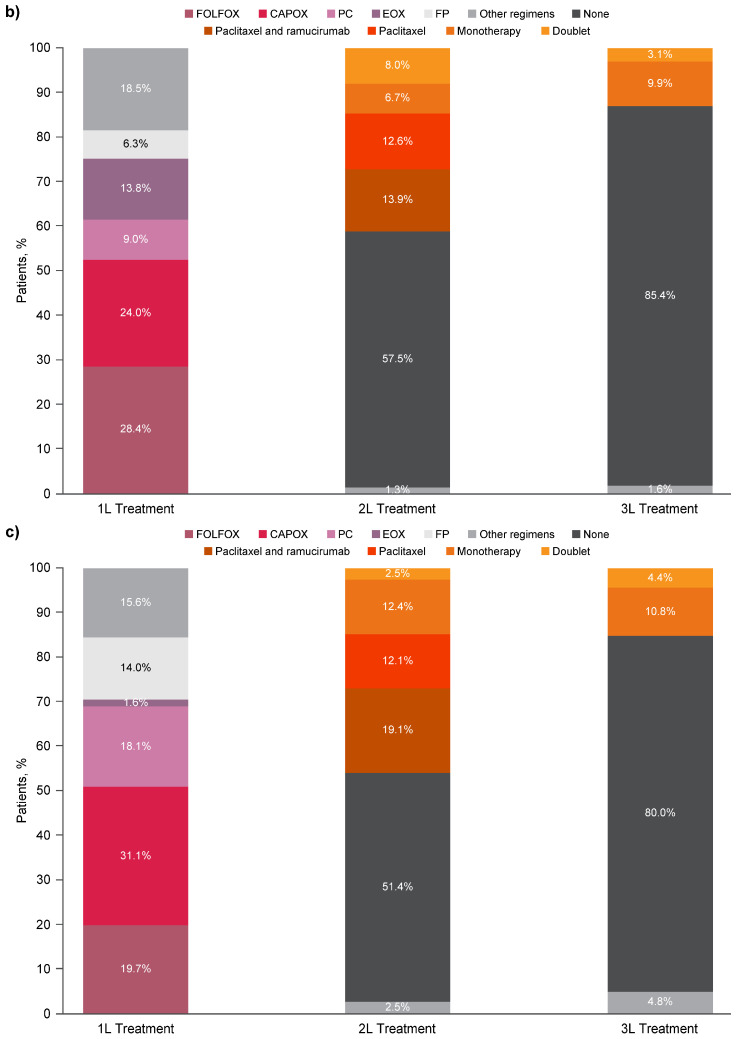
1L chemotherapy and 2L/3L treatment regimens for (**a**) all patients (*n* = 1357), (**b**) patients with HER2-negative disease (*n* = 951), and (**c**) patients with HER2-positive disease (*n* = 315). 1L, first-line; 2L, second-line; 3L, third-line; CAPOX, capecitabine and oxaliplatin; EOX, epirubicin, oxaliplatin, and capecitabine; FOLFOX, folinic acid, fluorouracil, and oxaliplatin; FP, fluorouracil and cisplatin; HER2, human epidermal growth factor receptor 2; PC, cisplatin and capecitabine.

As previously noted, a majority of patients (56.3%) received only one line of therapy; 27.6% of patients received two lines of therapy; and 16.1% received three lines of therapy. The most common 2L regimens during the 2015–2019 period were paclitaxel alone and paclitaxel in combination with ramucirumab (Figure 2a). Most patients who received third-line (3L) therapy were treated with monotherapy (Figure 2a). Similar trends for 2L and 3L treatment regimens were observed across HER2-negative and HER2-positive subgroups during the 2015–2019 period (Figure 2b,c).

### 3.5. PFS

**Figure 3:**
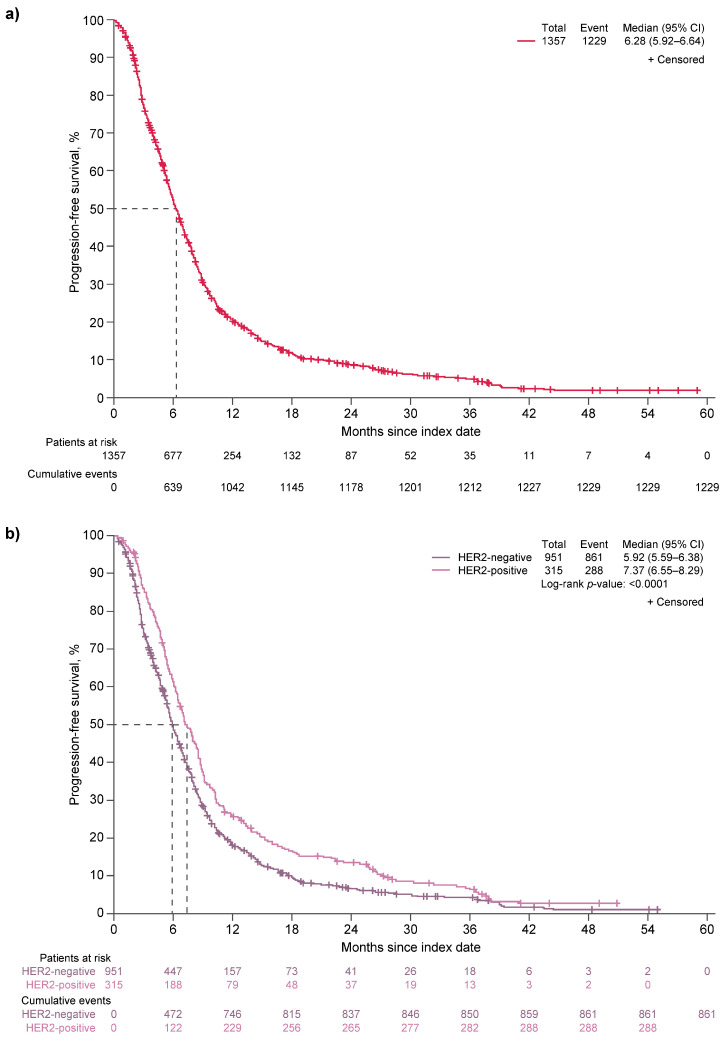
Kaplan–Meier plots of PFS (**a**) for all patients, all regimens (*n* = 1357), and (**b**) by HER2 status, all regimens (*n* = 1266). CI, confidence interval; HER2, human epidermal growth factor receptor 2; PFS, progression-free survival.

Median PFS for all patients was 6.28 months (95% CI, 5.92–6.64; Figure 3a). Patients with HER2-negative disease had significantly shorter median PFS compared with those who had HER2-positive disease (5.92 months [95% CI, 5.59–6.38] vs. 7.37 months [95% CI, 6.55–8.29]; *p* < 0.0001; Figure 3b).

### 3.6. OS

**Figure 4:**
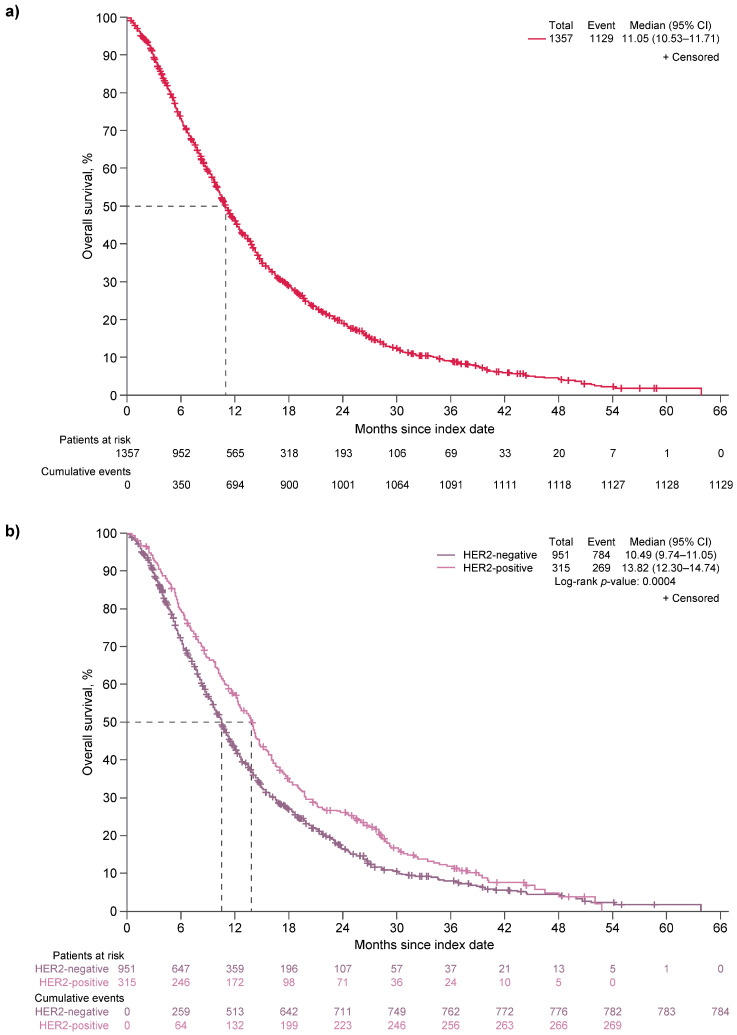
Kaplan–Meier plots of OS (**a**) for all patients, all regimens (*n* = 1357), and (**b**) by HER2 status, all regimens (*n* = 1266). CI, confidence interval; HER2, human epidermal growth factor receptor 2; OS, overall survival.

Median OS was 11.05 months for all patients (95% CI, 10.53–11.71; Figure 4a). Patients with HER2-negative disease had a significantly shorter median OS compared with those with HER2-positive disease (10.49 months [95% CI, 9.74–11.05] vs. 13.82 months [95% CI, 12.30–14.74]; *p* = 0.0004; Figure 4b).

Results from AFT models showed that patients with HER2-negative disease had significantly shorter OS than those with HER2-positive disease (unadjusted TR, 0.790; 95% CI, 0.701–0.890; *p* = 0.0001 (Table S1); adjusted TR [aTR], 0.812; 95% CI, 0.722–0.913; *p* = 0.0005 (Table S2)). In the adjusted AFT model, the overall *p*-value for each confounder was statistically significant. Prognostic factors associated with worse OS included diffuse vs. intestinal histological subtype (aTR, 0.838; 95% CI, 0.742–0.946); ≥4 vs. 0–3 metastatic sites (aTR, 0.749; 95% CI, 0.622–0.900); ECOG PS of 1 vs. 0 (aTR, 0.852; 95% CI, 0.754–0.963) and ECOG PS ≥ 2 vs. 0 (aTR, 0.507; 95% CI, 0.429–0.598); bone metastases (aTR, 0.783; 95% CI, 0.670–0.916); ascites (aTR, 0.744; 95% CI, 0.657–0.841); NLR 4 to <8 vs. <4 (aTR, 0.789; 95% CI, 0.704–0.885) and NLR ≥ 8 vs. <4 (aTR, 0.636; 95% CI, 0.539–0.750; Table S2).

### 3.7. Comparison of OS by HER2 Status Among Patients Receiving 1L FOLFOX

Median OS was significantly shorter for the 270 patients with HER2-negative disease receiving 1L FOLFOX compared with the 62 patients with HER2-positive disease receiving 1L FOLFOX (10.72 months [95% CI, 9.28–11.55] vs. 16.61 months [95% CI, 11.18–21.09]; *p* = 0.0009; Figure S1). Patients with HER2-negative disease had significantly shorter OS in the unadjusted AFT model (TR, 0.664; 95% CI, 0.520–0.848; *p* = 0.0010; Table S3) and after adjusting for confounders (aTR, 0.675; 95% CI, 0.525–0.868; *p* = 0.0022; Table S4) than patients with HER2-positive disease. In the adjusted AFT model, ECOG PS and NLR were significantly associated with OS. Prognostic factors associated with worse survival included ECOG PS of ≥2 vs. 0 (aTR, 0.619; 95% CI, 0.450–0.852), NLR 4 to < 8 vs. <4 (aTR, 0.731; 95% CI, 0.590–0.905), and NLR ≥8 vs. <4 (aTR, 0.633; 95% CI, 0.477–0.840).

### 3.8. Comparison of OS by HER2 Status Among Patients Receiving 1L CAPOX

Median OS did not significantly differ between the 228 patients with HER2-negative disease and 98 patients with HER2-positive disease who received 1L CAPOX (11.02 months [95% CI, 8.62–14.44] vs. 13.98 months [95% CI, 11.88–16.09]; *p* = 0.54; Figure S2). HER2-negative disease was not significantly associated with OS in the unadjusted or adjusted AFT analyses (TR = 0.888; 95% CI, 0.695–1.135; *p* = 0.34; Tables S5 and S6). In the adjusted AFT model, histological subtype, number of metastatic sites, ECOG PS, and NLR were significantly associated with OS. Prognostic factors associated with shorter OS were diffuse vs. intestinal histological subtype (aTR, 0.631; 95% CI, 0.487–0.817); ≥4 vs. 0–3 metastatic sites (aTR, 0.462; 95% CI, 0.311–0.687); ECOG PS of 1 vs. 0 (aTR, 0.686; 95% CI, 0.523–0.900) and ECOG PS ≥ 2 vs. 0 (aTR, 0.357; 95% CI, 0.238–0.536); NLR 4 to <8 vs. <4 (aTR, 0.609; 95% CI, 0.479–0.773) and NLR ≥ 8 vs. <4 (aTR, 0.642; 95% CI, 0.450–0.915; Table S6).

### 3.9. Number of Patients with HER2-Negative Disease Eligible for 1L Polychemotherapy

Using PSA, the average estimated number of patients with HER2-negative advanced G/GEJa in Spain who were eligible for 1L polychemotherapy in 2024 was 2856 (95% CI, 1619–4134; Figure S3).

## 4. Discussion

Advanced G/GEJa is associated with severe symptoms, significant healthcare resource utilization and cost, and poor prognosis, particularly for HER2-negative disease, which lacked targeted therapies before the approval of pembrolizumab and nivolumab for advanced G/GEJa [3,9]. This study aimed to describe the clinical characteristics, treatment patterns, and survival outcomes of patients in Spain with advanced G/GEJa; to compare PFS and OS by HER2 status; and to estimate the number of patients in Spain with HER2-negative advanced G/GEJa who were eligible for 1L polychemotherapy. A majority of patients (56.3%) received only one line of therapy; 27.6% received two lines; and 16.1% received three lines. FOLFOX and CAPOX were the most common 1L regimens from 2017 onward, and paclitaxel alone and paclitaxel in combination with ramucirumab were the most common 2L regimens. Median OS was similar with FOLFOX and CAPOX for patients with HER2-negative G/GEJa (10.72 vs. 11.02, respectively) but was longer with FOLFOX vs. CAPOX for patients with HER2-positive disease (16.61 vs. 13.98, respectively). Median PFS (6.28 months overall) was significantly shorter for patients with HER2-negative vs. HER2-positive disease (5.92 vs. 7.37 months). Median OS (11.05 months overall) was also significantly shorter for patients with HER2-negative vs. HER2-positive disease (10.49 vs. 13.82 months). In adjusted models for all 1L treatment regimens, prognostic factors associated with worse OS included HER2-negative vs. HER2-positive status; diffuse vs. intestinal histological subtype; ≥4 vs. 0–3 metastatic sites; ECOG PS of 1 or ≥2 vs. 0; bone metastases; ascites; NLR 4 to <8 vs. <4; and NLR ≥ 8 vs. <4.

In this cohort of patients, most had de novo disease, a low ECOG PS (0–1), and a low NLR (<4). Over half of patients in this study received only 1L treatment, perhaps reflecting delayed diagnosis and poor prognosis [36,37]. The emergence of FOLFOX and CAPOX as the most common 1L chemotherapy regimens between 2015 and 2019 is consistent with the European Society for Medical Oncology (ESMO) guidelines for gastric cancer, which recommend platinum-fluoropyrimidine doublet chemotherapy for 1L treatment of advanced G/GEJa [36]. Paclitaxel alone and paclitaxel in combination with ramucirumab as the most common 2L treatments also aligns with the most recent ESMO guidelines [36,38].

Consistent with previous estimates that approximately three-quarters of advanced G/GEJa cases are HER2-negative [10,26], 70.1% of patients in this study had HER2-negative disease. Compared with patients with HER2-positive disease, patients with HER2-negative disease were slightly younger and less likely to be male. They were also more likely to have primary gastric (vs. GEJ) tumors with diffuse (vs. intestinal) histology. Male predominance of HER2-positive gastric cancer has been noted previously [39,40,41], as have associations between HER2 overexpression and older age, GEJ primary tumor site, and intestinal histology [10,41,42].

The observed differences in survival between HER2-positive and HER2-negative subgroups (i.e., significantly shorter PFS and OS in patients with HER2-negative disease, and significantly shorter OS after adjusting for confounders in patients with HER2-negative disease, including those receiving 1L FOLFOX) may be mainly attributed to the lack of targeted therapies for patients with HER2-negative disease in the 1L setting during the time period studied (2015–2021). HER2 is a transmembrane receptor tyrosine kinase that is activated by heterodimerization with other members of the human epidermal growth factor receptor family, initiating signal transduction pathways that stimulate proliferation, differentiation, and survival of tumor cells [43]. Targeted therapy for HER2-positive disease, including antibodies that inhibit HER2-mediated signaling by reducing HER2 receptor expression or dimerization (e.g., trastuzumab, which was received by 92.7% of patients with HER2-positive disease as part of their 1L treatment regimen), has contributed to improved survival in that population [13,43]. The differences between HER2-positive and HER2-negative populations may increase further with the advent of trastuzumab deruxtecan [15,16].

In 2021, the immune checkpoint inhibitors nivolumab and pembrolizumab were approved in Europe for 1L treatment of adults with HER2-negative advanced or metastatic gastric, GEJ, or esophageal adenocarcinoma whose tumors express PD-L1 with a CPS ≥ 5 for nivolumab and ≥10 for pembrolizumab [21,22]. The approval for pembrolizumab was updated in 2023 to allow a CPS ≥ 1 [44]; however, in Spain, pembrolizumab financing is restricted to patients whose tumors express PD-L1 with a CPS ≥ 10 [45]. There remains an unmet need for personalized targeted therapy to improve survival outcomes in patients with HER2-negative G/GEJa [36], as not all G/GEJa tumors express PD-L1 [23,24,25] or enough PD-L1 to receive financing (CPS ≥ 5 for nivolumab or CPS ≥ 10 for pembrolizumab in Spain [46]). Furthermore, the estimated 2856 patients with HER2-negative advanced G/GEJa in Spain who were eligible for 1L treatment with polychemotherapy in 2024 represent a substantial proportion of the total number of new cases of G/GEJ cancer in Spain in 2024 (6868 patients), underscoring this unmet need.

This study has several strengths. It is a large-scale, real-world study based on the AGAMENON-SEOM registry database, with broad representation of regions across Spain. The study design allowed for each patient to have at least 2 years of follow-up. The data reflect evolving trends in contemporary disease management over recent years, including shifts in polychemotherapy regimens. Altogether, the study provides detailed, up-to-date information on a large cohort of patients with advanced G/GEJa in Spain.

This study also has limitations. First, the treatment landscape has changed markedly since the introduction of immunotherapies and later treatment lines in HER2-positive disease (trastuzumab deruxtecan). Second, it is retrospective and therefore subject to potential for bias and missing information. Third, it is not a population-based study because the AGAMENON-SEOM registry only includes centers that opt to participate. Access to 2L and 3L treatment may have been limited in some centers. Fourth, there was a minority of patients (6.7%) whose HER2 status was unknown. Fifth, it is not known how many of the patients receiving polychemotherapy would also be suitable for new agents combined with polychemotherapy. When estimating the number of patients with HER2-negative advanced G/GEJa who were eligible for 1L polychemotherapy, there was no single data source representative of patients in Spain with HER2-negative advanced G/GEJa that included all of the required parameters; therefore, multiple sources of data were used for this estimation. To address this limitation, PSA was used to quantify the uncertainty of each parameter and estimate the number of eligible patients.

## 5. Conclusions

This study is based on a robust analysis of the largest cohort of patients with advanced G/GEJa in Spain, describing clinical characteristics, treatment patterns, and survival outcomes. Comparisons of PFS and OS between patients with HER2-negative and HER2-positive disease provide further evidence that there is a treatment gap for HER2-negative disease, which represents the majority (approximately three-quarters [10,26]) of advanced G/GEJa cases. Irrespective of HER2 status, the aggressiveness of this disease is apparent in the minority of patients who receive subsequent lines of therapy beyond 1L treatment. More effective treatments are critically needed. Findings from this study may help inform new healthcare policies and spur further research toward improving disease management and treatment outcomes.

## Figures and Tables

**Table 1:** Patient eligibility for the AGAMENON-SEOM registry and this study.

	Inclusion Criteria	Exclusion Criteria
AGAMENON-SEOM registry	• Histologically confirmed advanced gastric, GEJ, or distal esophageal adenocarcinoma • Age ≥ 18 years at diagnosis • Received at least 1 cycle of polychemotherapy (combination chemotherapy using 2 or more drugs) as 1L treatment • Patients who received 1L treatment within a clinical trial are included, if the treatment received included chemotherapy, regardless of the additional drug • Followed for at least 3 months after treatment initiation, except those with an early death during this period • Data from regular clinical practice, with at least 3 months of follow-up • Provided written informed consent, if alive at time of enrollment	• Participating in a clinical trial with no standard chemotherapy, regardless of the association of the molecularly targeted therapy • Previous systemic therapy for advanced gastric, GEJ, or distal esophageal adenocarcinoma • Less than 6 months elapsed from the last recorded date of prior treatment (neoadjuvant or adjuvant chemotherapy, radiotherapy, or chemoradiotherapy) • Therapy regimen not coded • Epithelial cell carcinoma and squamous cell and esophageal cancers, or any cancer other than G/GEJa
Study	• Met all inclusion criteria for AGAMENON-SEOM registry • Initiated 1L polychemotherapy for advanced G/GEJa during the index period (1 January 2015 to 31 December 2019) • Received treatment in Spain	• Met any exclusion criteria for AGAMENON-SEOM registry • Distal esophageal cancer (primary site)

1L, first-line; GEJ, gastroesophageal junction; G/GEJa, gastric/gastroesophageal junction adenocarcinoma.

**Table 2:** Sociodemographic and disease characteristics.

Characteristic	All Patients ^a^ (*n* = 1357)	HER2-Negative (*n* = 951)	HER2-Positive (*n* = 315)
Age at 1L treatment initiation			
Median (25th percentile, 75th percentile)	64.7 (55.5, 72.0)	63.8 (54.6, 71.5)	65.8 (56.9, 72.4)
Minimum–maximum	19.9–88.5	19.8–88.5	22.9–86.3
Age < 65 years	693 (51.1)	510 (53.6)	144 (45.7)
Male sex	913 (67.3)	615 (64.7)	238 (75.6)
BMI (kg/m^2^)			
Underweight: BMI < 18.5	84 (6.2)	65 (6.8)	16 (5.1)
Normal weight: BMI 18.5 to <25	731 (53.9)	525 (55.2)	154 (48.9)
Overweight: BMI 25 to <30	388 (28.6)	265 (27.9)	101 (32.1)
Obese: BMI ≥ 30	153 (11.3)	96 (10.1)	44 (14.0)
Missing	1 (0.1)	0 (0.0)	0 (0.0)
Number of comorbid conditions			
Median (25th percentile, 75th percentile)	0 (0, 1)	0 (0, 1)	0 (0, 1)
Minimum–maximum	0–6	0–6	0–4
≥1 comorbid condition	610 (45.0)	430 (45.2)	135 (42.9)
Chronic cardiopathy	160 (11.8)	115 (12.1)	33 (10.5)
ECOG PS			
0	315 (23.2)	225 (23.7)	68 (21.6)
1	845 (62.3)	590 (62.0)	198 (62.9)
2	188 (13.9)	131 (13.8)	45 (14.3)
3 or 4	9 (0.7)	5 (0.5)	4 (1.3)
Primary tumor site			
GEJ	373 (27.5)	225 (23.7)	129 (41.0)
Gastric	972 (71.6)	719 (75.6)	183 (58.1)
Not available	12 (0.9)	7 (0.7)	3 (1.0)
Tumor stage, locally advanced unresectable, without metastasis	93 (6.9)	68 (7.2)	17 (5.4)
De novo G/GEJa	1147 (84.5)	777 (81.7)	288 (91.4)
Surgery of the primary tumor	338 (24.9)	272 (28.6)	44 (14.0)
Lauren histological type			
Intestinal	504 (37.1)	295 (31.0)	186 (59.0)
Diffuse	522 (38.5)	432 (45.4)	58 (18.4)
Mixed	57 (4.2)	43 (4.5)	10 (3.2)
Not available/not classifiable	274 (20.2)	181 (19.0)	61 (19.4)
Signet ring cells			
Not present	693 (51.1)	442 (46.5)	214 (67.9)
Present, <50%	74 (5.5)	64 (6.7)	9 (2.9)
Present, ≥50%	125 (9.2)	107 (11.3)	9 (2.9)
Present, unknown percentage	232 (17.1)	195 (20.5)	21 (6.7)
Not available	233 (17.2)	143 (15.0)	62 (19.7)
Histological grade			
Grade 1	117 (8.6)	62 (6.5)	53 (16.8)
Grade 2	326 (24.0)	211 (22.2)	101 (32.1)
Grade 3	580 (42.7)	460 (48.4)	87 (27.6)
Not available	334 (24.6)	218 (22.9)	74 (23.5)
Number of metastatic sites			
0 or 1	657 (48.4)	483 (50.8)	125 (39.7)
2	409 (30.1)	282 (29.7)	106 (33.7)
3	176 (13.0)	115 (12.1)	50 (15.9)
≥4	115 (8.5)	71 (7.5)	34 (10.8)
Metastatic site			
Locoregional lymph nodes	859 (63.3)	561 (59.0)	241 (76.5)
Peritoneum	659 (48.6)	505 (53.1)	106 (33.7)
Non-locoregional lymph nodes	600 (44.2)	406 (42.7)	159 (50.5)
Liver	447 (32.9)	255 (26.8)	165 (52.4)
Ascites	320 (23.6)	244 (25.7)	47 (14.9)
Lung	180 (13.3)	97 (10.2)	70 (22.2)
Bone	158 (11.6)	114 (12.0)	33 (10.5)
Other	168 (12.4)	110 (11.6)	41 (13.0)
NLR ^b^			
Median (25th percentile, 75th percentile)	3.4 (2.2, 5.1)	3.3 (2.2, 4.9)	3.6 (2.2, 5.8)
Minimum–maximum	0.3–102.0	0.3–102.0	0.8–22.3
NLR category			
Low: <4	819 (60.4)	595 (62.6)	172 (54.6)
Moderate: 4 to <8	370 (27.3)	237 (24.9)	102 (32.4)
High: ≥8	142 (10.5)	97 (10.2)	38 (12.1)
Not available	26 (1.9)	22 (2.3)	3 (1.0)

Counts and percentages are shown for categorical variables. Median (25th, 75th percentile) and minimum–maximum are shown for noncategorical measures. 1L, first-line; BMI, body mass index; ECOG PS, Eastern Cooperative Oncology Group performance status; GEJ, gastroesophageal junction; G/GEJa, gastric/gastroesophageal junction adenocarcinoma; HER2, human epidermal growth factor receptor 2; NLR, neutrophil-to-lymphocyte ratio. ^a^ 91 patients had unknown HER2 status. ^b^ 26 patients did not have NLR data available (*n* = 22, patients with HER2-negative disease; *n* = 3, patients with HER2-positive disease; *n* = 1, unknown HER2 status).

**Table 3:** Treatment characteristics.

Characteristic	All Patients ^a^ (*n* = 1357)	HER2-Negative (*n* = 951)	HER2-Positive (*n* = 315)
Year initiated 1L treatment			
2015	335 (24.7)	238 (25.0)	64 (20.3)
2016	281 (20.7)	202 (21.2)	58 (18.4)
2017	303 (22.3)	219 (23.0)	68 (21.6)
2018	249 (18.3)	155 (16.3)	78 (24.8)
2019	189 (13.9)	137 (14.4)	47 (14.9)
1L chemotherapy regimen			
FOLFOX	355 (26.2)	270 (28.4)	62 (19.7)
CAPOX	345 (25.4)	228 (24.0)	98 (31.1)
PC	154 (11.3)	86 (9.0)	57 (18.1)
EOX	146 (10.8)	131 (13.8)	5 (1.6)
FP	107 (7.9)	60 (6.3)	44 (14.0)
Other doublet	186 (13.7)	124 (13.0)	46 (14.6)
Other triplet	64 (4.7)	52 (5.5)	3 (1.0)
1L treatment duration, months ^b^			
Median (25th percentile, 75th percentile)	4.8 (2.8, 7.2)	4.6 (2.6, 6.9)	5.2 (3.6, 8.5)
Minimum–maximum	0.2–39.7	0.2–39.7	0.4–37.9
Number of 1L chemotherapy cycles ^c^			
Median (25th percentile, 75th percentile)	6 (4, 11)	6 (4, 11)	7 (5, 12)
Minimum–maximum	1–75	1–60	1–58
Number of treatment lines			
1	764 (56.3)	547 (57.5)	162 (51.4)
2	374 (27.6)	265 (27.9)	90 (28.6)
3	219 (16.1)	139 (14.6)	63 (20.0)
2L treatment regimen			
None	764 (56.3)	547 (57.5)	162 (51.4)
Paclitaxel and ramucirumab	202 (14.9)	132 (13.9)	60 (19.0)
Paclitaxel	165 (12.2)	120 (12.6)	38 (12.1)
Ramucirumab	6 (0.4)	2 (0.2)	4 (1.3)
Other monotherapy	110 (8.1)	62 (6.5)	35 (11.1)
Doublet	90 (6.6)	76 (8.0)	8 (2.5)
Immunotherapy	8 (0.6)	6 (0.6)	2 (0.6)
Other regimen	12 (0.9)	6 (0.6)	6 (1.9)
3L treatment regimen			
None	1138 (83.9)	812 (85.4)	252 (80.0)
Monotherapy	139 (10.2)	94 (9.9)	34 (10.8)
Doublet	49 (3.6)	30 (3.2)	14 (4.4)
Immunotherapy	2 (0.1)	1 (0.1)	1 (0.3)
Other regimen	29 (2.1)	14 (1.5)	14 (4.4)

Counts and percentages are shown for categorical variables. Median (25th, 75th percentile) and minimum–maximum are shown for noncategorical measures. 1L, first-line; 2L, second-line; 3L, third-line; CAPOX, capecitabine and oxaliplatin; EOX, epirubicin, oxaliplatin, and capecitabine; FOLFOX, folinic acid, fluorouracil, and oxaliplatin; FP, fluorouracil and cisplatin; HER2, human epidermal growth factor receptor 2; PC, cisplatin and capecitabine. ^a^ 91 patients had unknown HER2 status. ^b^ For patients receiving polychemotherapy: if the chemotherapy medications had different treatment durations, the longest treatment duration is reported. ^c^ For patients receiving polychemotherapy: if the chemotherapy medications had different numbers of cycles, the highest number of cycles is reported.

## Data Availability

The AGAPONIS study is a collaborative study between Astellas and AGAMENON group, both of which contributed to the creation of the study protocol. The data for this publication are from the AGAMENON registry (https://www.agamenonstudy.com/), which is property of SEOM (Spanish Society of Medical Oncology) exclusively.

## Supplementary Materials

The following supporting information can be downloaded at: https://www.mdpi.com/article/10.3390/cancers17132164/s1, Figure S1: Kaplan–Meier plot of OS by HER2 status among patients receiving 1L FOLFOX; Figure S2: Kaplan–Meier plot of OS by HER2 status among patients receiving 1L CAPOX; Figure S3: PSA to estimate the number of patients with HER2-negative advanced G/GEJa in Spain who were eligible for 1L polychemotherapy in 2024; Table S1: Unadjusted AFT model of OS for patients with HER2-negative and HER2-positive disease: all regimens (*n* = 1266); Table S2: Adjusted AFT model of OS for patients with HER2-negative and HER2-positive disease: all regimens (*n* = 1266); Table S3: Unadjusted AFT model of OS for patients with HER2-negative and HER2-positive disease: 1L FOLFOX (*n* = 332); Table S4: Adjusted AFT model of OS for patients with HER2-negative and HER2-positive disease: 1L FOLFOX (*n* = 332); Table S5: Unadjusted AFT model of OS for patients with HER2-negative and HER2-positive disease: 1L CAPOX (*n* = 326); Table S6: Adjusted AFT model of OS for patients with HER2-negative and HER2-positive disease: 1L CAPOX (*n* = 326).

## Abbreviations

The following abbreviations are used in this manuscript:

1L	First-line
2L	Second-line
3L	Third-line
AFT	Accelerated failure time
aTR	Adjusted time ratio
BMI	Body mass index
CAPOX	Capecitabine and oxaliplatin
CI	Confidence interval
CPS	Combined positive score
ECOG PS	Eastern Cooperative Oncology Group performance status
EOX	Epirubicin, oxaliplatin, and capecitabine
ESMO	European Society for Medical Oncology
FOLFOX	Folinic acid, fluorouracil, and oxaliplatin
FP	Fluorouracil and cisplatin
GEJ	Gastroesophageal junction
G/GEJa	Gastric/gastroesophageal junction adenocarcinoma
HER2	Human epidermal growth factor receptor 2
NLR	Neutrophil-to-lymphocyte ratio
OS	Overall survival
PC	Cisplatin and capecitabine
PD-1	Programmed death receptor 1
PD-L1	Programmed death-ligand 1
PFS	Progression-free survival
PSA	Probabilistic sensitivity analysis
SE	Standard error
SEOM	Spanish Society of Medical Oncology
TR	Time ratio
