# Supplementary material for: Treatment and Outcomes in Advanced Gastroesophageal Adenocarcinoma in the Pre-Immunotherapy Era Based on the Spanish AGAMENON-SEOM Registry
Source: Cancers (Basel). 2025 Jun 27;17(13):2164. doi: 10.3390/cancers17132164 (PMC12248623; doi:10.3390/cancers17132164)
Supplement: Supplementary file 1 [file cancers-17-02164-s001.zip › cancers-3646044-supplementary.pdf]

Supplementary Materials

Treatment and Outcomes in Advanced Gastroesophageal Adenocarcinoma in the Pre-Immunotherapy Era Based on the Spanish AGAMENON-SEOM Registry

Paula Jimenez-Fonseca, Alberto Carmona-Bayonas, Jaime Álvarez-Cañada, Amy Storfer-Isser, Marta Martin-Richard, Tamara Sauri, Juana María Cano, Elia Martínez Moreno, Pablo Pérez-Wert, Javier López, Francisco Garcia Navalon, Lucía Gómez-González, Maribel Ruiz Martín, Ana Belén Rupérez Blanco, Flora López-López, Emilse Roncancio-Díaz, Belén Corbacho, Marta Mateo, Paloma Anguita-Alonso and Javier Gallego Plazas

Table S1. Unadjusted AFT model of OS for patients with HER2-negative and HER2-positive disease: all regimens (*n* = 1266).

Variable	*n*	Events	Estimate	SE	Time Ratio	95% CI	*p*-value
HER2							0.0001
HER2-positive (reference)	315	269					
HER2-negative	951	784	−0.236	0.061	0.790	0.701–0.890	

AFT, accelerated failure time; CI, confidence interval; HER2, human epidermal growth factor receptor 2; OS, overall survival; SE, standard error.

Table S2. Adjusted AFT model of OS for patients with HER2-negative and HER2-positive disease: all regimens (n = 1266).

Variable	*n*	Events	Estimate	SE	Time Ratio	95% CI	*p*-value
HER2							0.0005
HER2-positive (reference)	315	269					
HER2-negative	951	784	−0.208	0.060	0.812	0.722–0.913	
Lauren histological subtype							0.0261
Intestinal (reference)	481	395					
Diffuse	490	415	−0.177	0.062	0.838	0.742–0.946	
Mixed	53	41	−0.152	0.130	0.859	0.666–1.109	
Not available	242	202	−0.144	0.070	0.866	0.755–0.994	
Number of metastatic sites							0.0021
0–3 (reference)	1161	961					
≥4	105	92	−0.290	0.094	0.749	0.622–0.900	
ECOG PS							<0.0001
0 (reference)	293	223					
1	788	660	−0.160	0.062	0.852	0.754–0.963	
≥2	185	170	−0.680	0.085	0.507	0.429–0.598	
Bone metastases							0.0022
No (reference)	1119	922					
Yes	147	131	−0.244	0.080	0.783	0.670–0.916	
Ascites							<0.0001
No (reference)	975	800					
Yes	291	253	−0.296	0.063	0.744	0.657–0.841	
NLR							<0.0001
<4 (reference)	767	613					
4 to <8	339	297	−0.237	0.058	0.789	0.704–0.885	
≥8	135	121	−0.453	0.084	0.636	0.539–0.750	
Missing	25	22	−0.111	0.177	0.895	0.632–1.267	

AFT, accelerated failure time; CI, confidence interval; ECOG PS, Eastern Cooperative Oncology Group performance status; HER2, human epidermal growth factor receptor 2; NLR, neutrophil-to-lymphocyte ratio; OS, overall survival; SE, standard error.

Table S3. Unadjusted AFT model of OS for patients with HER2-negative and HER2-positive disease: 1L FOLFOX (*n* = 332).

**Variable**	***n***	**Events**	**Estimate**	**SE**	**Time Ratio**	**95% CI**	***p*-value**
HER2							0.0010
HER2-positive (reference)	62	51					
HER2-negative	270	218	−0.409	0.125	0.664	0.520–0.848	

1L, first-line; AFT, accelerated failure time; CI, confidence interval; FOLFOX, folinic acid, fluorouracil, and oxaliplatin; HER2, human epidermal growth factor receptor 2; OS, overall survival; SE, standard error.

Table S4. Adjusted AFT model of OS for patients with HER2-negative and HER2-positive disease: 1L FOLFOX (n = 332).

Variable	*n*	Events	Estimate	SE	Time Ratio	95% CI	*p*-value
HER2							0.0022
HER2-positive (reference)	62	51					
HER2-negative	270	218	−0.393	0.129	0.675	0.525–0.868	
Lauren histological subtype							0.9948
Intestinal (reference)	111	94					
Diffuse	142	115	−0.001	0.117	0.999	0.794–1.258	
Mixed	15	11	0.058	0.242	1.060	0.660–1.702	
Not available	64	49	−0.011	0.140	0.989	0.752–1.301	
Number of metastatic sites							0.7783
0–3 (reference)	308	249					
≥4	24	20	−0.055	0.194	0.947	0.647–1.385	
ECOG PS							<0.0001
0 (reference)	52	37					
1	200	159	0.007	0.145	1.007	0.758–1.337	
≥2	80	73	−0.480	0.163	0.619	0.450–0.852	
Bone metastases							0.0550
No (reference)	282	227					
Yes	50	42	−0.268	0.140	0.765	0.582–1.006	
Ascites							0.0828
No (reference)	244	196					
Yes	88	73	−0.195	0.112	0.823	0.660–1.026	
NLR							0.0033
<4 (reference)	178	133					
4 to <8	101	89	−0.313	0.109	0.731	0.590–0.905	
≥8	49	44	−0.457	0.144	0.633	0.477–0.840	
Missing	4	3	−0.057	0.449	0.944	0.392–2.276	

1L, first-line; AFT, accelerated failure time; CI, confidence interval; ECOG PS, Eastern Cooperative Oncology Group performance status; FOLFOX, folinic acid, fluorouracil, and oxaliplatin; HER2, human epidermal growth factor receptor 2; NLR, neutrophil-to-lymphocyte ratio; OS, overall survival; SE, standard error.

Table S5. Unadjusted AFT model of OS for patients with HER2-negative and HER2-positive disease: 1L CAPOX (*n* = 326).

Variable	*n*	Events	Estimate	SE	Time Ratio	95% CI	*p*-value
HER2							0.3437
HER2-positive (reference)	98	86					
HER2-negative	228	179	−0.119	0.125	0.888	0.695–1.135	

1L, first-line; AFT, accelerated failure time; CAPOX, capecitabine and oxaliplatin; CI, confidence interval; HER2, human epidermal growth factor receptor 2; OS, overall survival; SE, standard error.

Table S6. Adjusted AFT model of OS for patients with HER2-negative and HER2-positive disease: 1L CAPOX (*n* = 326).

Variable	*n*	Events	Estimate	SE	Time Ratio	95% CI	*p*-value
HER2							0.3491
HER2-positive (reference)	98	86					
HER2-negative	228	179	−0.116	0.124	0.890	0.698–1.135	
Lauren histological subtype							0.0014
Intestinal (reference)	149	119					
Diffuse	101	85	−0.461	0.132	0.631	0.487–0.817	
Mixed	13	9	−0.177	0.288	0.838	0.476–1.475	
Not available	63	52	−0.410	0.143	0.664	0.502–0.878	
Number of metastatic sites							0.0001
0–3 (reference)	298	239					
≥4	28	26	−0.773	0.202	0.462	0.311–0.687	
ECOG PS							<0.0001
0 (reference)	70	50					
1	217	181	−0.377	0.138	0.686	0.523–0.900	
≥2	39	34	−1.030	0.207	0.357	0.238–0.536	
Bone metastases							0.9952
No (reference)	287	230					
Yes	39	35	−0.001	0.167	0.999	0.720–1.386	
Ascites							0.5252
No (reference)	264	212					
Yes	62	53	−0.094	0.148	0.910	0.681–1.216	
NLR							0.0003
<4 (reference)	204	154					
4 to <8	86	77	−0.496	0.122	0.609	0.479–0.773	
≥8	30	29	−0.444	0.181	0.642	0.450–0.915	
Missing	6	5	−0.211	0.398	0.810	0.371–1.766	

1L, first-line; AFT, accelerated failure time; CAPOX, capecitabine and oxaliplatin; CI, confidence interval; ECOG PS, Eastern Cooperative Oncology Group performance status; HER2, human epidermal growth factor receptor 2; NLR, neutrophil-to-lymphocyte ratio; OS, overall survival; SE, standard error.

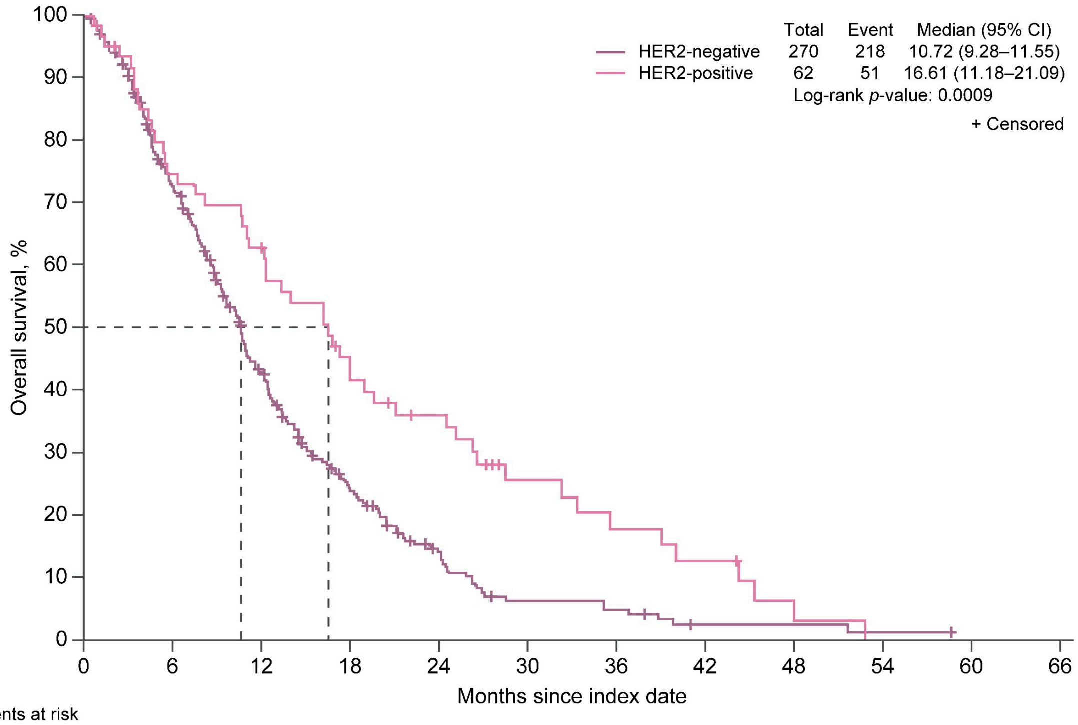

	0	6	12	18	24	30	36	42	48	54	60
**Patients at risk**											
HER2-negative	270	183	96	47	23	9	7	2	2	1	0
HER2-positive	62	44	37	24	18	10	7	5	2	0	
**Cumulative events**											
HER2-negative	0	70	142	182	199	212	214	217	217	218	218
HER2-positive	0	15	22	33	37	42	45	47	49	51	

1L, first-line; CI, confidence interval; FOLFOX, folinic acid, fluorouracil, and oxaliplatin; HER2, human epidermal growth factor receptor 2; OS, overall survival.

Figure S1. Kaplan–Meier plot of OS by HER2 status among patients receiving 1L FOLFOX.

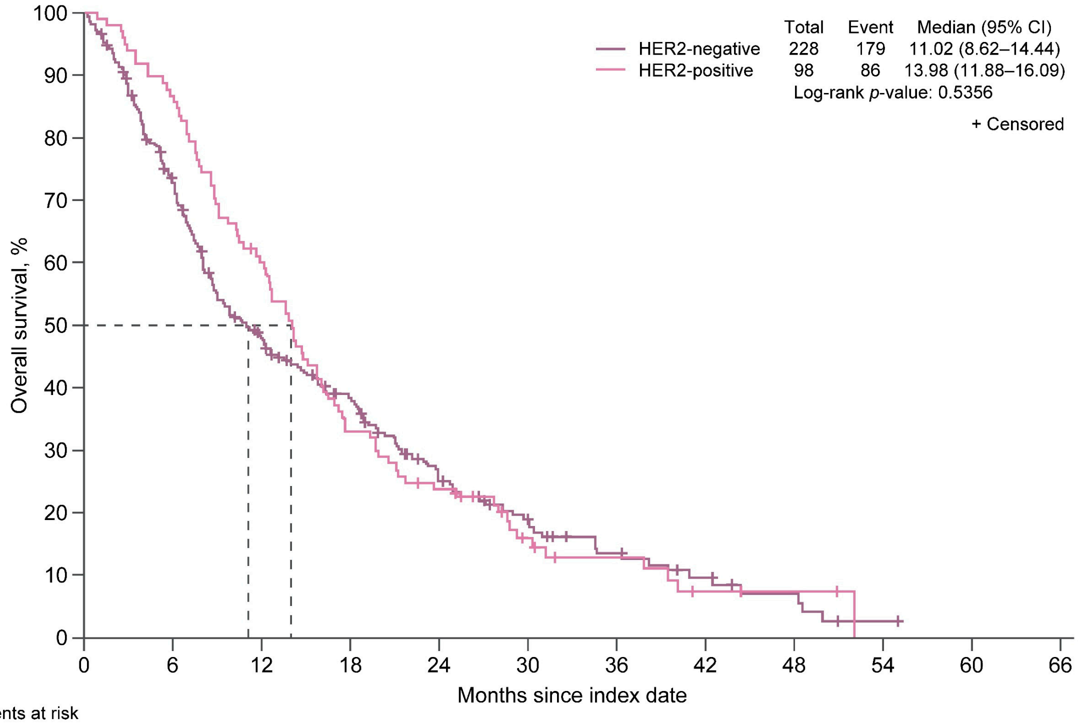

	0	6	12	18	24	30	36	42	48	54	60
**Patients at risk**											
HER2-negative	228	157	97	70	41	27	16	9	5	1	0
HER2-positive	98	85	58	32	22	11	7	3	2	0	
**Cumulative events**											
HER2-negative	0	59	113	131	154	163	170	174	176	179	179
HER2-positive	0	13	39	65	74	80	82	85	85	86	

1L, first-line; CAPOX, capecitabine and oxaliplatin; CI, confidence interval; HER2, human epidermal growth factor receptor 2; OS, overall survival.

Figure S2. Kaplan–Meier plot of OS by HER2 status among patients receiving 1L CAPOX.

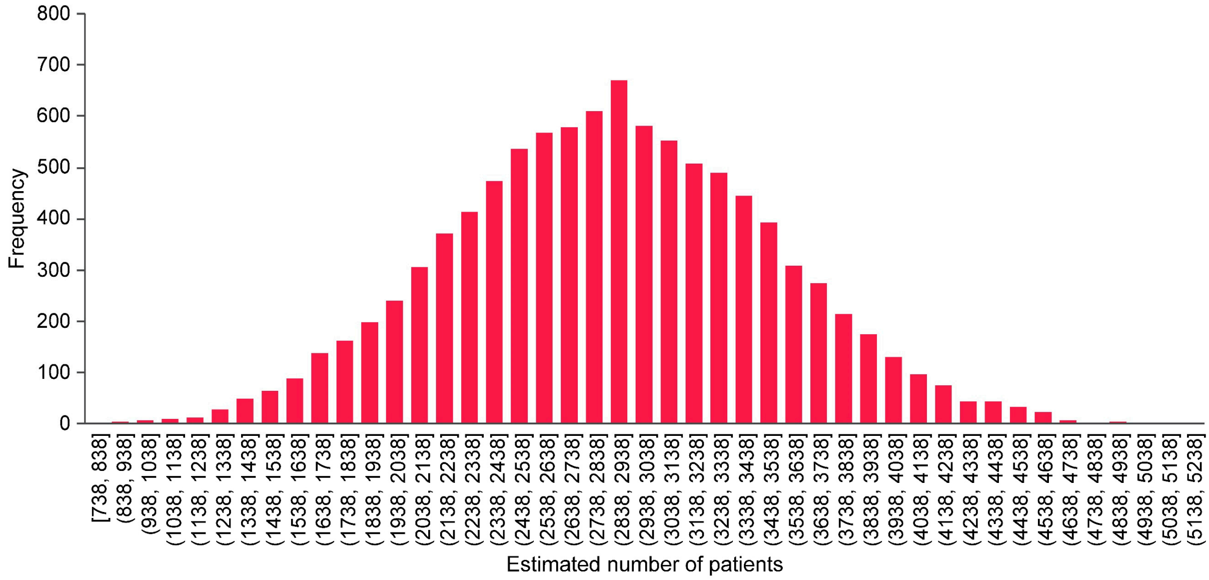

1L, first-line; G/GEJa, gastric/gastroesophageal junction adenocarcinoma; HER2, human epidermal growth factor receptor 2; PSA, probabilistic sensitivity analysis.

Figure S3. PSA to estimate the number of patients with HER2-negative advanced G/GEJa in Spain who were eligible for 1L polychemotherapy in 2024.
